# The IgLON Family Member Negr1 Promotes Neuronal Arborization Acting as Soluble Factor via FGFR2

**DOI:** 10.3389/fnmol.2015.00089

**Published:** 2016-01-13

**Authors:** Francesca Pischedda, Giovanni Piccoli

**Affiliations:** ^1^Department of Medical Biotechnology and Translational Medicine, Università degli Studi di MilanoMilano, Italy; ^2^Department of Neuroscience, Istituto Di Neuroscienze-Consiglio Nazionale delle Ricerche, San Raffaele Scientific ParkMilano, Italy

**Keywords:** adhesion protein, neuron development, ADAM10, Negr1, FGFR2

## Abstract

IgLON proteins are GPI anchored adhesion molecules that control neurite outgrowth. In particular, Negr1 down-regulation negatively influences neuronal arborization *in vitro* and *in vivo*. In the present study, we found that the metalloprotease ADAM10 releases Negr1 from neuronal membrane. Ectodomain shedding influences several neuronal mechanisms, including survival, synaptogenesis, and the formation of neurite trees. By combining morphological analysis and virus-mediated selective protein silencing in primary murine cortical neurons, we found that pharmacologically inhibition of ADAM10 results in an impairment of neurite tree maturation that can be rescued upon treatment with soluble Negr1. Furthermore, we report that released Negr1 influences neurite outgrowth in a P-ERK1/2 and FGFR2 dependent manner. Together our findings suggest a role for Negr1 in regulating neurite outgrowth through the modulation of FGFR2 signaling pathway. Given the physiological and pathological role of ADAM10, Negr1, and FGFR2, the regulation of Negr1 shedding may play a crucial role in sustaining brain function and development.

## Introduction

Neurite outgrowth is a fundamental process that allows the establishment of functional wiring in the developing brain. This process can be summarized as a mechanism where extracellular cues bind to transmembrane receptors and trigger signaling cascades which eventually reorganize neuronal structure (Raper and Mason, 2010). Therefore, understanding how neuronal projections calibrate their responses to developmental cues might provide critical information about how the brain builds functional connectivity. Several families of transmembrane or membrane proteins, such as semaphorin, neuroligin, neurexin, and immuno-globulin super families, have been implicated in the regulation of neurite formation (Maness and Schachner, 2007). IgLONs are adhesion protein belonging to the immunoglobulin superfamily. IgLON members are highly glycosylated proteins, possess three C2-type Ig-like domains and bind to the lipid membrane by a GPI-anchor (Miyata et al., 2003). Accumulating evidence indicates that IgLON are tightly implicated in neuronal functions such as synaptic formation, plasticity, and neurite tree development (Gil et al., 1998; Schäfer et al., 2005; Hashimoto et al., 2008, 2009; Pischedda et al., 2014; Sanz et al., 2015). In particular, it has been suggested that each IgLON protein can promote or inhibit neuronal maturation depending on the particular complement of IgLON expressed at the surface (Hashimoto et al., 2009). IgLON proteins may form homophilic and heterophilic complexes at the cell surface or with juxtaposed cells to modulate adhesion and neurite outgrowth. For example LSAMP facilitates the extension of neurites from neurons expressing LSAMP while it inhibits branch formation in neurons that do not express LSAMP (Mann et al., 1998). Furthermore, it has been demonstrated that two IgLON members, neurotrimin (Gil et al., 1998), and LSAMP (Zhukareva et al., 1997) can be released and act to regulate neurite outgrowth. We have previously described the role of the IgLON family member Negr1 in regulating neurite arborization (Pischedda et al., 2014). In this previous study, we observed that neurons lacking endogenous Negr1 display a clear morphological phenotype -with a reduction of dendrite length and number compared to wild type cells- suggesting that Negr1 positively modulates neuronal tree growth. The mechanism underlying Negr1 impact on neuron morphology, however, remains obscure. Strikingly, it has been recently demonstrated that IgLONs family members, including Negr1 can be shed from the surface of cortical neurons through a metalloprotease dependent proteolytic pathway (Sanz et al., 2015). In the study reported here, we found that the metalloprotease ADAM10 cleaves Negr1 from neuronal membrane and characterized the impact of ADAM10-Negr1 on neuronal structural development. In particular, we found that released Negr1 influences neuronal morphology by activating a pathway that requires FGFR2 receptor and ERK1/2 phosphorylation.

## Materials and methods

### Lentiviral vector constructs, virus production, and plasmids

Negr1 and FGFR2 target sequences were identified, synthesized, and cloned into GFP-expressing pLVTH, as previously described (Zhou et al., 2006; Bauer et al., 2009; Pischedda et al., 2014). In brief, oligonucleotides (oligo-nt) coding for a 5′-pseudoBglII-site, a sense-oligo-nt-loop-antisense-oligo-nt, transcription termination site, and a 3′pseudo-XbaI-restriction-site were purchased from Metabion. Sense and antisense oligos were annealed and subsequently phosphorylated. The fragments were cloned 3′ to the H1-promoter of pBC KS+(ClaI)-H1, resulting in pBC KS+(ClaI)-sh. The H1sh cassettes were isolated with ClaI, blunted, and cloned into the blunted ClaI/BamH1 site of pLV transfer-vector. pLV is a modified plasmid transfer vector derived from original pLVTH (Wiznerowicz and Trono, 2003), in which the BamH1-tetO-H1-ClaI fragment was excised. All recombinant lentiviruses were produced by transient transfection of HEK293T cells according to standard protocols (Wiznerowicz and Trono, 2003). Primary neuronal cultures were transduced with viruses at multiplicity of infection (MOI) 3 if not otherwise specified. mNegr1 cDNA (Addgene clone C3342IRCKp5014P057-rzpdm13–21) was cloned into strep-FLAG pcDNA3.1 vector (Gloeckner et al., 2009).

### Negr1 purification and deglycosylation

For protein purification, HEK293 cells transfected with strep-FLAG Negr1 were lysed in RIPA buffer (150 mM NaCl, 50 mM HEPES, 0.5% NP40, 1% Sodium-deoxycholate) for 1 h at 4°C and then processed for streptavidin immunoprecipitation. Protein were eluted from STREP resin in elution buffer (2.5 mM Desthiobiotin, 100 mM Tris-HCL, 150 mM NaCl, 1 mM EDTA) in mild agitation for 1 h at 4°C. The protein concentration was measured via standard Bradford assay (Bio-Rad) and protein purity was assessed by SDS-PAGE followed by silver staining. Where indicated, after purification strep-FLAG Negr1 was treated with PNGase F (5 units, 20 min, 37°C; Sigma-Aldrich, Germany) and then analyzed by western blotting.

### Neuronal cultures and drugs

Cortical neuron cultures were prepared from mouse embryos (E17.5–18.5; strain C57BL/6). High-density (750–1000 cells/mm^2^) and medium-density (150–200 cells/mm^2^) neuronal cultures were plated and grown on 6-well plastic tissue culture plates (Iwaki; Bibby Sterilin Staffordshire, UK) or on 12 mm diameter coverslips put into 24-well plastic tissue culture plates as previously described (Iwaki; Bibby Sterilin; Piccoli et al., 2007). In these cultures glial growth is reduced to less than 0.5% of the nearly pure neuronal population (Brewer et al., 1993). Neurons were treated daily with FGFb 20 ng/ml, FGF7/KGF 20 ng/ml, IGF 5 ng/ml (all from Peprotech, NJ USA) or every second day with GI 254023X 20 μM (TOCRIS Bioscience, UK) or MEK inhibitor U0126 100 nM (Sigma-Aldrich). Strep-FLAG-Negr1, 40 ng/ml, was performed at DIV10. The National and Institutional Animal committees have approved all the procedures involving animal present in this work (IACUC 625).

### Biochemistry and antibody

Neurons were washed in PBS and lysed in RIPA buffer (150 mM NaCl, 50 mM HEPES, 0.5% NP40, 1% Sodium-deoxycholate). After 1 h under mild agitation, lysate was clarified by centrifugation for 20 min at 16,000*g*. All procedures were performed at 4°C. Protein sample were measured via standard Bradford assay (Bio-Rad. USA). For protein identification and relative quantification via Western blotting, a proper volume of sample containing an equal amount of proteins was diluted with 0.25% 5X Laemmli buffer and loaded onto 10% SDS-PAGE gels; the proteins were transferred onto nitrocellulose membrane (Sigma-Aldrich) at 80 V for 120 min at 4°C.

The primary antibodies were applied overnight in a blocking buffer (20 mM Tris, pH 7.4, 150 mM NaCl, 0.1% Tween 20, and 5% nonfat dry milk); primary antibodies (source in parentheses) included rabbit anti- FGFR2 1:200 (Santa Cruz Biotechnology, USA), goat anti-Negr1 1:1000 (R&D, USA), mouse anti-NCAM 1:1000 (BD Biosciences, USA), rabbit anti-GFP 1:5000 (Life Technology, USA), mouse anti-Actin 1:2000 (Sigma-Aldrich), rabbit anti RpS6 1:2000, rabbit anti-p42/44(pERK), and rabbit anti-42/44 (ERK; Cell Signaling, USA). The secondary antibodies (HRP-conjugated anti-mouse, anti-rabbit, or anti-rat; Jackson ImmunoResearch, UK) were used in a ratio of 1:8000. The signal was detected using an ECL detection system (GE Healthcare). Films were acquired on a GS-800 densitometer (BioRad) calibrated following the manufacturers' instructions, and protein abundance was estimated as a function of the optical density of a specific band quantified by ImageJ software (NIH). Unless otherwise stated, all the other chemicals were purchased from Applichem, Germany.

### Immunofluorescence and quantification

Neuronal cultures were infected with viruses at days *in vitro* (DIV) 1–2 or transfected at DIV4 with Lipofectamine 2000 following manifacture's protocol (Life Technology). For the immunostaining experiments, neurons were fixed at DIV 18 in 4% paraformaldehyde and 4% sucrose at room temperature. GFP positive neurons were randomly chosen for quantification. The fluorescence images were acquired in blind using a LSM Zeiss 510 confocal microscope with Zeiss 63X objective (Karl Zeiss, Jena, Germany) at a resolution of 2048 × 2048 pixels, pixel size = 0.098 mm. All the measurements were performed in blind using NeuronStudio (available at http://research.mssm.edu/cnic/tools.html). Neurites were automatically traced and quantified by the software in terms of length, number and morphology (Wearne et al., 2005; Rodriguez et al., 2008). In our analysis neurite number represents the total number of segments. Data were then logged and analyzed in Microsoft Excel.

### Statistical analysis

All data are expressed as mean ± SEM. Data were analyzed with an unpaired Student's *t*-test (two classes) or ANOVA followed by Tuckey's *post hoc*-test (more than two classes). The indication level of significance (p) is indicated for specific experiments throughout the text and in Supplementary Table 1. For the analysis of neurons morphology in the different experimental condition we considered at least 12 cells randomly chosen in at least four experiments.

## Results

### Negr1 modulates neurite tree

**Figure 1:**
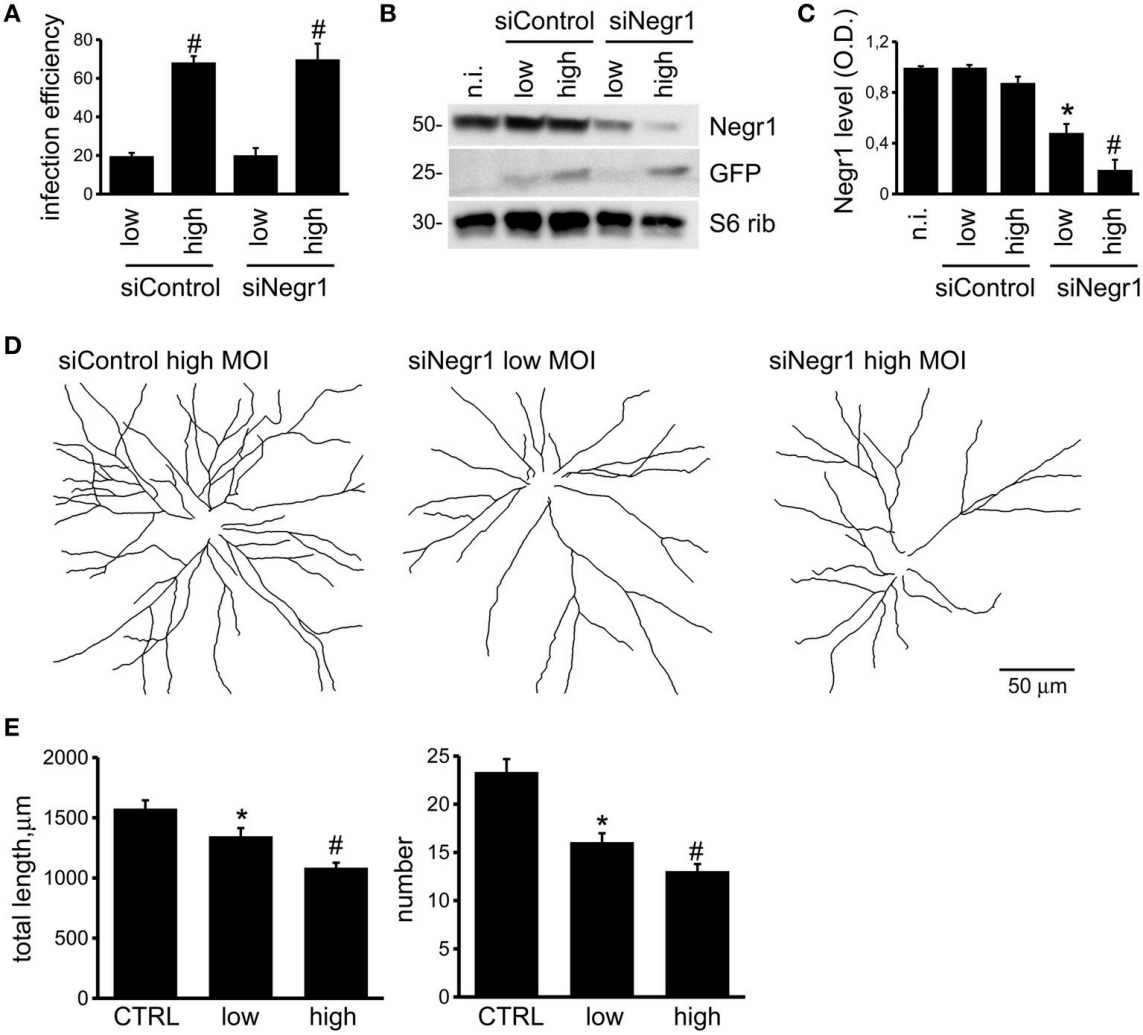
**Negr1 influences neuritic tree formation**. Cortical neurons were infected at DIV4 with low (MOI = 0.3) or high (MOI = 3) titer of viruses expressing GFP and scramble siRNA (siControl) or siRNA against Negr1 (siNegr1). Infection with low or high viral titer resulted in different infection efficiency, evaluated as fraction of GFP positive cells within the entire population, stained with DAPI. Data are expressed as mean ± SEM, *n* = 4. ^*^p < 0.05 vs. not infected (n.i.), #*p* < 0.05 vs. low infection **(A)**. Cells were solubilized and then assassed by immuno-blotting at DIV18 **(B)**. The graph reports the optical density of the band relative to Negr1 protein, normalized vs. S6 ribosomial protein (S6 rib) value. Data are expressed as mean ± SEM, *n* = 4. ^*^p < 0.05 vs. not infected (n.i.), #*p* < 0.05 vs. low infection **(C)**. Neurons were infected at DIV4 and processed for immunofluorescence at DIV18. Panels show camera lucida tracing **(D)**. Graphs show neurite total length and number **(E)**. Data are reported as mean ± SEM; ^*^p < 0.01 vs. siControl (CTRL), #*p* < 0.05 vs. low infection. Scale bar = 50 μm.

Recent evidence suggested that IgLONs members, including Negr1, are released into the extracellular space via metalloproteinase and that metalloproteinase activity on IgLONs has an impact on neuronal maturation (Sanz et al., 2015). We have previously showed that Negr1 influences neuritic tree development (Pischedda et al., 2014). In the light of these findings, Negr1 may influence neuronal morphology acting *in cis* as a membrane bound protein and/or *in trans* as a soluble protein. To investigate these two scenarios, we infected cortical neurons at DIV4 with viruses expressing GFP marker together with either Negr1 specific siRNA (siNegr1) or scramble control (siControl) following two experimental paradigms: (1) cultures infected with a high viral titer (MOI = 3); (2) cultures infected with a low viral titer (MOI = 0.3). In both cases, cultures were processed for subsequent investigation at DIV18. Biochemical and imaging analysis showed that the titer of infection influenced the extent of both infection efficiency and Negr1 protein reduction (Figures 1A–C). Next we studied the morphology of GFP positive neurons in the two different conditions. Interestingly we reported that cultures exposed to high viral titer show a more pronounced reduction in terms of neurite number and length than cultures exposed to low viral titer (Figures 1D–E, Supplementary Figure 3A, and Table 1). These data suggest that the overall amount of Negr1 expressed by cells influences neuronal morphology.

**Table 1:** **The table reports neurite total length and number for each experimental condition investigated**.

**Infection**	**Treatment**	**Total length ± S.E**.	**Number ± S.E**.
siControl	Not treated	1587.15±58.67	23.47±1.04
siNegr1	Low MOI	1347±57.3	16.06±0.93
siNegr1	High MOI	1084.82±41.54	14±0.66
siControl	rNegr1	2043.5±76.98	30.42±1.26
siControl	GI 254023X	590.2±42.72	10.1±0.55
siControl	GI 254023X + rNegr1	1169.7±90.17	19.5±1.96
siNegr1	rNegr1	1673.64±71.23	25.61±1.09
siControl	U0126	787.05±58.94	12.95±0.8
siControl	U0126 + rNegr1	742.7±43.63	11±0.62
siControl	FGFb	1654.43±57.1	25.43±1.06
siControl	FGF7	2105±84.93	31.5±1.18
siControl	IGF	1612.18±87.14	26.06±1.19
siNegr1	FGFb	1505.88±64.04	22.54±1.03
siNegr1	FGF7	1686.14±62.18	26.86±0.9
siNegr1	IGF	1398.91±62.28	19.23±0.98
siFGFR2	Not treated	1032.15±121.67	16.35±1.66
siFGFR2	rNegr1	945.82±117.68	16.68±1.81

*Data are shown as mean ± SEM*.

### Negr1 shedding by ADAM10 modulates neurite tree formation

**Figure 2:**
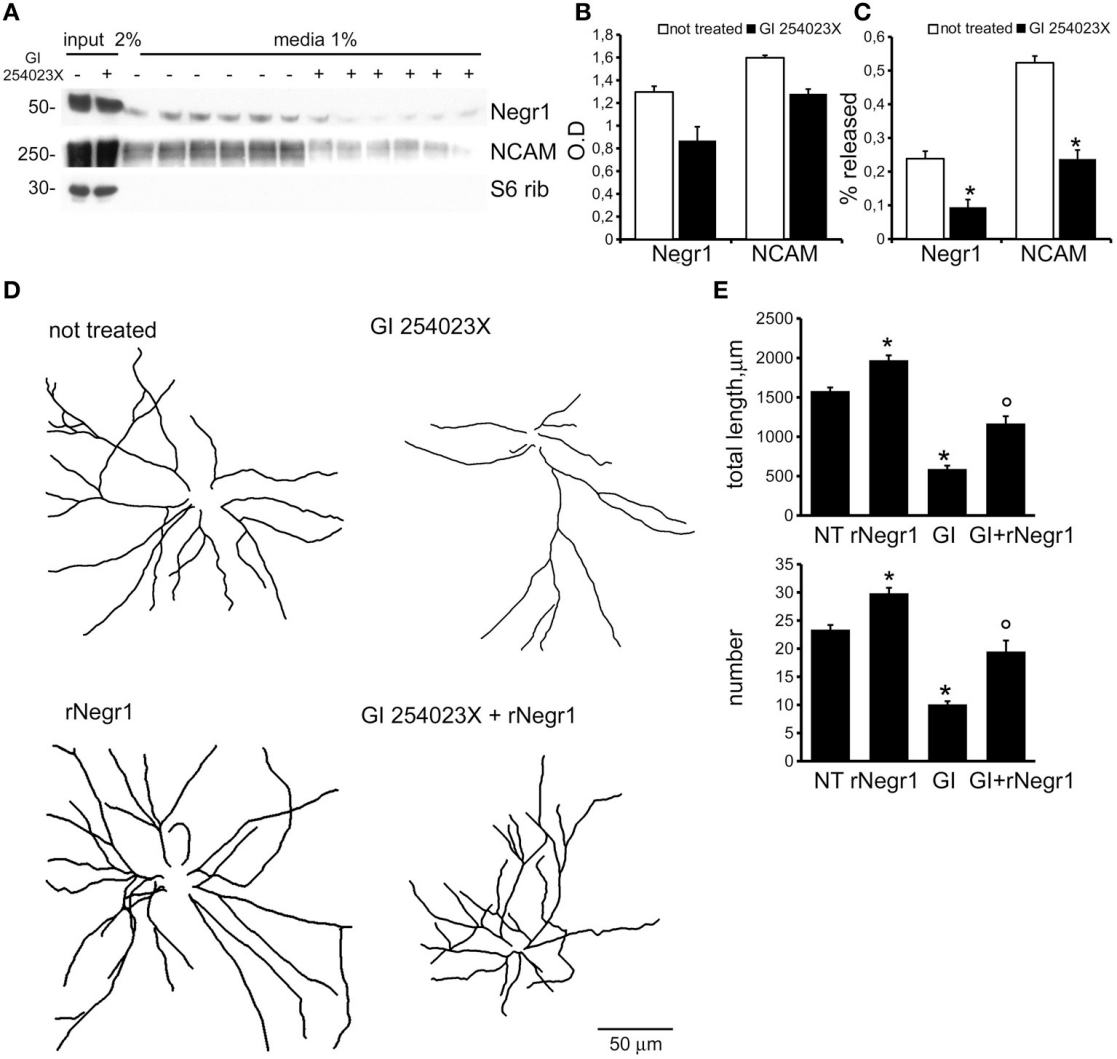
**ADAM10 activity modulates neuritic tree development**. Cortical neurons were infected at DIV4 with siControl and then treated every second day with DMSO (20 μM, not treated) or with ADAM10 inhibitor GI 254023X (20 μM) from DIV10 to DIV18. Cells and relative conditioned media were assayed for western-blotting to measure NCAM, Negr1, and S6 ribosomial protein level **(A)**. Graphs show NCAM, Negr1 level in the cellular lysate expressed as optical density, normalized vs. and S6 ribosomial protein (S6 rib) value **(B)** and the extent of Negr1 and NCAM release in media expressed as percentage of the relative amount measured in cellular lysate **(C)**. Data are reported as mean ± SEM; *n* = 6, ^*^p < 0.05 vs. not treated. Cortical neurons were infected with siControl virus at DIV4 and treated every second day from DIV10 to DIV18 with DMSO (not treated, NT) or with ADAM10 inhibitor GI 254023X (20 μM, GI) and/or recombinant Negr1 (40 ng/ml, single administration at DIV 10, rNegr1). Neurons were processed for immunofluorescence at DIV18 and GFP positive neurons imaged via confocal microscopy. Panels show camera lucida tracing **(D)**. Graphs show neurite total length and number **(E)**. Data are reported as mean ± SEM; ^*^p < 0.001 vs. not treated (NT); °*p* 0.001 vs. GI 254023X (GI). Scale bar = 50 μm.

To confirm the biological relevance of Negr1 as soluble factor, we investigated in detail the presence of Negr1, NCAM, and S6 ribosomial protein, a well-established cytoplasmic marker, in samples prepared from neuronal culture at DIV18 or from the relative conditioned media. As expected, we detected all three proteins in the cellular lysate, but only Negr1 and NCAM in the media. It is well established that NCAM can be released by shedding via ADAM10 (Brennaman et al., 2014). Interestingly, when we treated neurons from DIV10 to DIV18 with the well characterized ADAM10 inhibitor, GI 254023X (20 μM, every second day), we noticed a robust reduction of the fraction of NCAM and Negr1 protein released in the media (Figures 2A–C). These experiments suggest that Negr1 can be shed from neuronal membrane by ADAM10. Sanz and colleagues previously demonstrated that metalloproteinase inhibitors severely impair neuronal morphological development in an IgLON dependent manner (Sanz et al., 2015). Consequently, we studied the morphological phenotype in cortical cultures treated with DMSO or with the ADAM inhibitor GI 254023X (20 μM, every second day) from DIV10 to 18. Analysing the incidence of pyknotic nuclei suggested that GI 254023X did not induce overt toxicity (Supplementary Figures 2A,B). Notably, we observed that chronic inhibition of ADAM reduced the total number of neurite as well as the number of first and second order neurite (Figures 2D–E, Supplementary Figures 1, 3B and Table 1). ADAM10 is involved in processing (and shedding) several membrane proteins implicated in neurite outgrowth, including NCAM, N-Cadherin, and L1-NCAM (Mechtersheimer et al., 2001; Jorissen et al., 2010; Paudel et al., 2013; Brennaman et al., 2014). We therefore sought to assess the direct contribution of soluble Negr1 to the regulation of neuron morphology *via* ADAM10. To achieve this, we purified 2XStrep-FLAG epitope tagged Negr1 (rNegr1) from transfected HEK293 cell using streptavidin resin. Protein purity was monitored by silver-staining (Supplementary Figure 2C). Negr1 is highly and specifically glycosylated *in vivo* (Miyata et al., 2003). Upon immuno-blot analysis, we detected rNegr1 as a band with an apparent molecular weight (MW) of 50 KDa, corresponding to the glycosylated form of Negr1. Upon incubation with the N-linked deglycosilase PNGase, we detected a protein band with an apparent MW of 38 KDa, corresponding to the MW predicted for unmodifed Negr1 (Expasy database, entry: Q80Z24; Supplementary Figure 2D). Thus, exogenous expression of rNegr1 resulted in a protein that possesses some of the molecular features of endogenous Negr1. Purified rNegr1 was administered to either DMSO or ADAM10 inhibitor treated cortical neuron at DIV10 at concentration of 40 ng/ml. Cells were fixed at DIV18 and processed for immunofluorescence. We observed that rNegr1 treatment influenced positively the morphology of ADAM10 inhibited neurons. Furthermore, rNegr1 treatment was associated to an increase of neurite number and length in DMSO treated neurons (Figures 2D,E, Supplementary Figures 1, 3B and Table 1). Taken together, these data support a role for soluble Negr1 released by ADAM10 in the modulation of neuronal morphology.

### Soluble Negr1 influences neuron morphology acting via FGFR2

**Figure 3:**
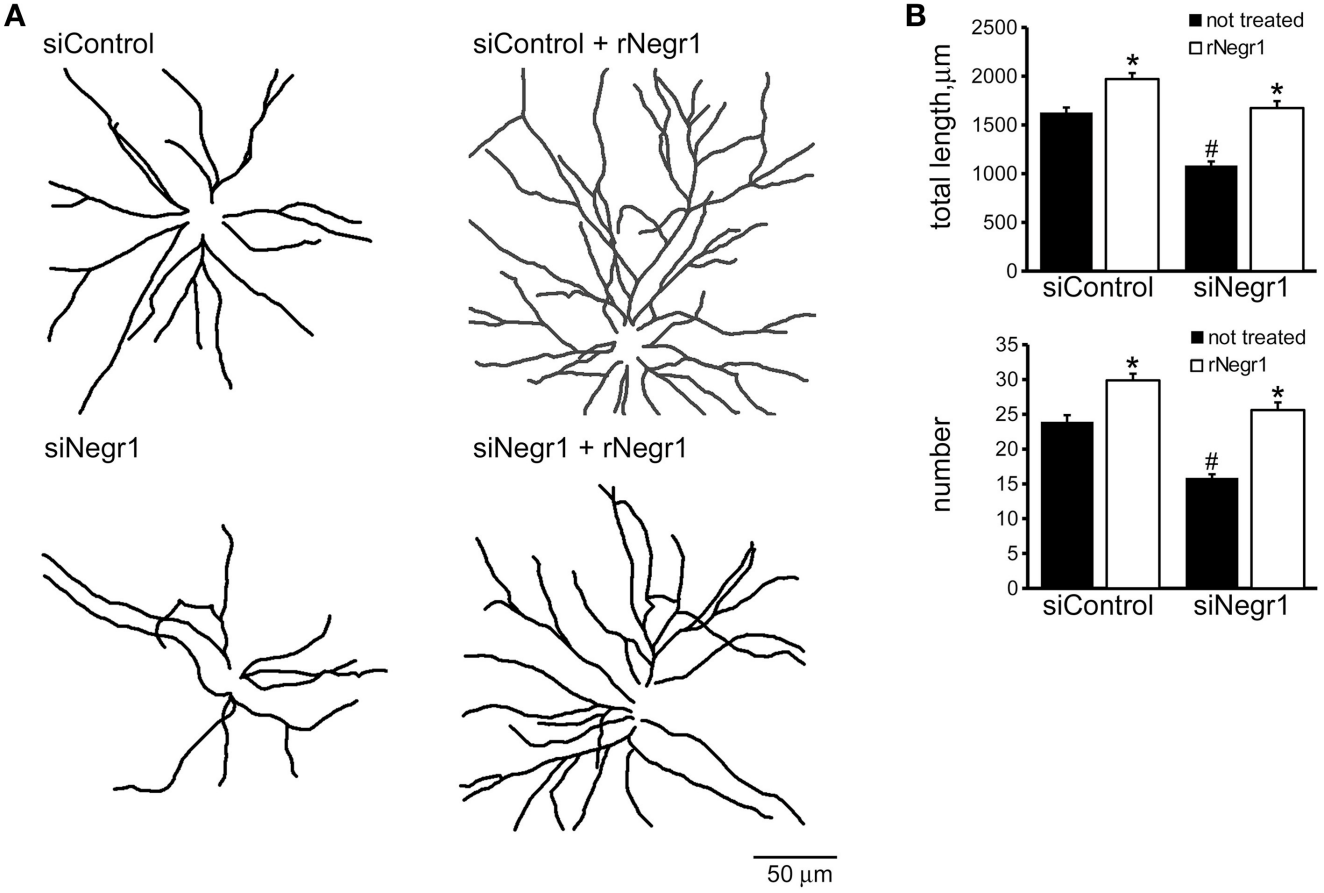
**Homodimerization is not essential to execute rNegr1 morphological effect**. Cortical neurons were infected with siControl or siNegr1 virus at DIV4 and treated with recombinant Negr1 (40 ng/ml, single administration at DIV 10, rNegr1). Neurons were processed for immunofluorescence at DIV18 and infected GFP positive neurons imaged via confocal microscopy. Panels show camera lucida tracing **(A)**. Graphs show neurite total length and number **(B)**. Data are reported as mean ± SEM; ^*^p < 0.001 vs. not treated, same infection, #*p* < 0.001 vs. siControl, same treatment. Scale bar = 50 mm.

**Figure 4:**
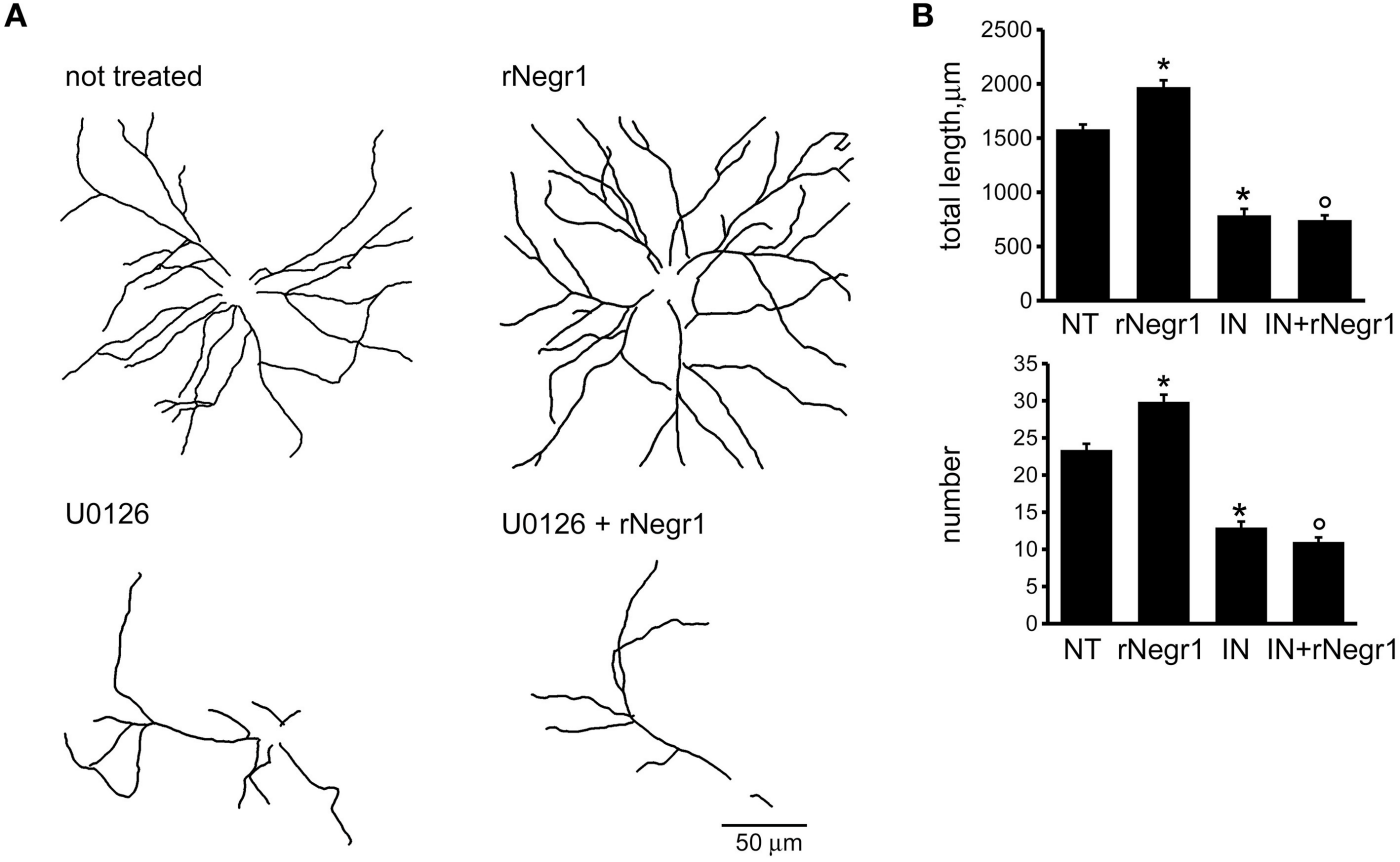
**MEK inhibition abolishes morphological effect of Negr1**. Cortical neurons were infected with siControl virus at DIV4 and treated every second day from DIV10 to DIV18 with DMSO or with MEK inhibitor U0126 (100 nM, IN) and/or recombinant Negr1 (40 ng/ml, single administration at DIV 10, rNegr1). Neurons were processed for immunofluorescence at DIV18 and infected GFP positive neurons imaged via confocal microscopy. Panels show camera lucida tracing **(A)**. Graphs show neurite total length and number **(B)**. Data are reported as mean ± SEM; ^*^p < 0.001 vs. not treated (NT), °*p* < 0.001 vs. rNegr1. Scale bar = 50 μm.

**Figure 5:**
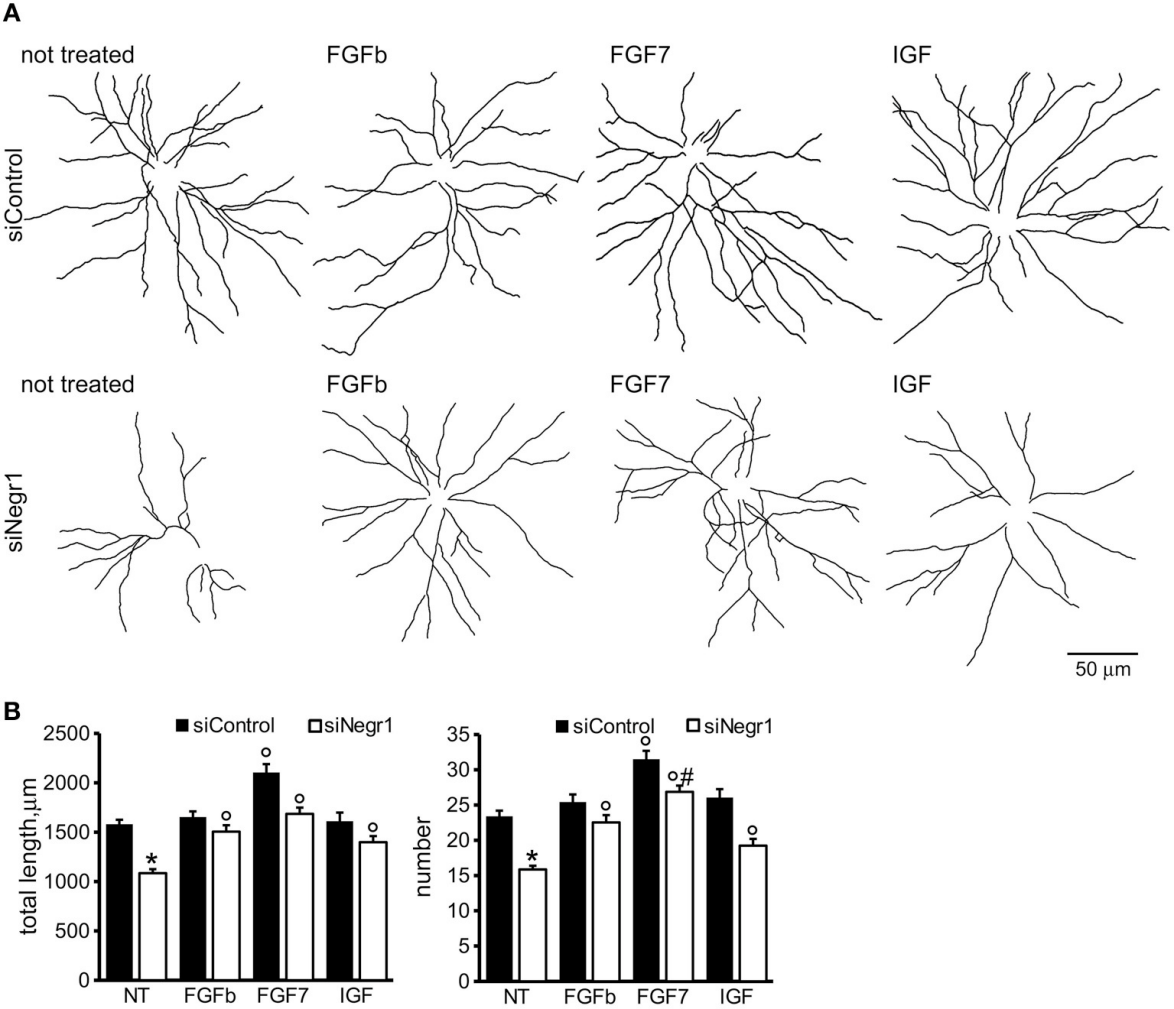
**FGFR2 activation ameliorates morphological effect due to Negr1 down-regulation**. Cortical neurons were infected with siControl or siNegr1 virus at DIV4 and treated daily from DIV10 to DIV18 with IGF (5 ng/ml), FGFb (20 ng/ml), and FGF7 (20 ng/ml). Neurons were processed for immunofluorescence at DIV18 and infected GFP positive neurons imaged via confocal microscopy. Panels show camera lucida tracing **(A)**. Graphs show neurite total length and number **(B)**. Data are reported as mean ± SEM; ^*^p < 0.001 vs. siControl, same treatment, °*p* < 0.001 vs. not treated, same infection (NT), #*p* < 0.01 vs. FGFb or IGF, same infection. Scale bar = 50 μm.

**Figure 6:**
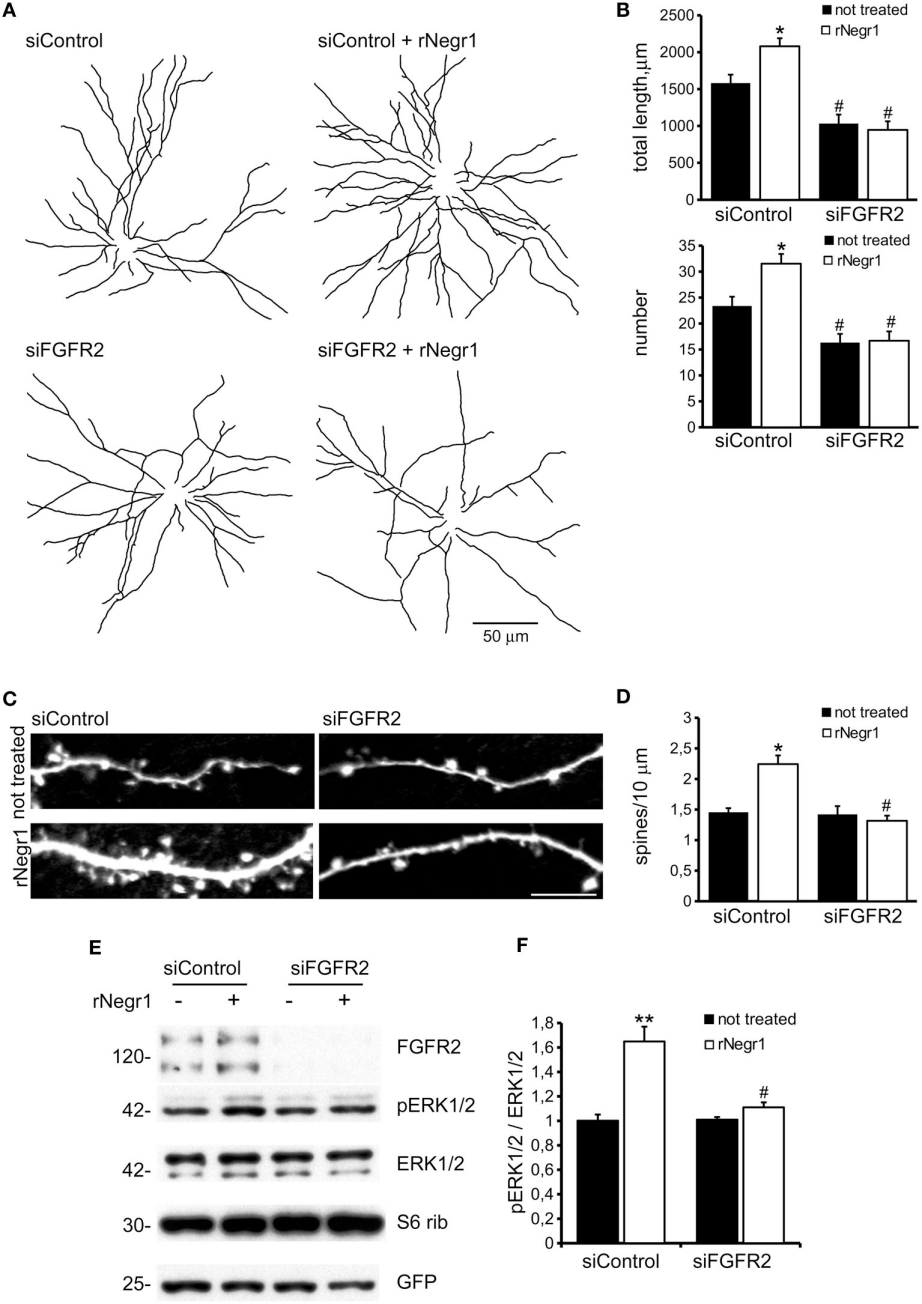
**Negr1 modulates neuron morphology via FGFR2**. Cortical neurons were infected at DIV4 with virus expressing siControl or siRNA against FGFR2 (siFGR2) and treated at DIV10 with recombinant Negr1 (40 ng/ml, rNegr1). Neurons were processed for immunofluorescence at DIV18 and infected GFP positive neurons imaged via confocal microscopy. Panels show camera lucida tracing **(A)**. Graphs show neurite total length and number **(B)**. Data are reported as mean ± SEM; ^*^p < 0.001 vs. not treated, same infection, #*p* < 0.01 vs. siControl, same treatment. Scale bar = 50 μm. Dendritic spines were recognized as mushroom like protrusions decorating the neurites **(C)**. Spine density was calculated as protrusion number along 10 μm of neuritic length. Data are reported as mean ± SEM; ^*^p < 0.001 vs. not treated, same infection, #*p* < 0.01 vs. siControl, same treatment. Scale bar = 10 μm **(D)**. Cortical neurons were infected with virus expressing siControl or siRNA against FGFR2 (siFGR2) and treated with recombinant Negr1 (40 ng/ml, rNegr1 10 min). Neurons were processed for western-blotting to investigate ERK1/2 phosphorylation **(E)**. The graph reports p-ERK1/2 level normalized vs. total ERK1/2 amount **(F)**. Data are expressed as mean ± SEM *n* = 5. ^**^p < 0.01 vs. not treated, same infection; #*p* < 0.01 vs. siControl, same treatment.

Biochemical studies suggest that IgLON proteins can form stable homodimers (Miyata et al., 2003). We therefore set out to test whether soluble rNegr1 executes its function *via* binding endogenous Negr1 expressed on neuronal membrane. To address this, we investigated the impact of soluble rNegr1 on cortical neuronal cultures where endogenous Negr1 expression had been down-regulated. Cortical neurons were infected at DIV4 with viruses expressing GFP together with Negr1 specific siRNA (siNegr1) or scramble siRNA (siControl) and treated at DIV10 with soluble rNegr1. As already observed, the abolishment of endogenous Negr1 expression correlated with a reduction of neuritic tree complexity (Pischedda et al., 2014). Intriguingly, the morphological effect of rNegr1 was evident also in Negr1 silenced neurons. In fact, rNegr1 treatment was able to rescue the morphological phenotype resulting from Negr1 silencing (Figure 3, Supplementary Figure 4A, and Table 1). This evidence suggests that the formation of a Negr1 homodimer is not strictly required for the morphological effect associated with the exposure to soluble Negr1. Instead, soluble Negr1 may associate with other proteins to influence neurite outgrowth. The ERK1/2 pathway had been recognized as a key player in neurite outgrowth (Maness and Schachner, 2007). We therefore sought to test if soluble Negr1 could influence neuronal morphology *via* the ERK1/2 signaling pathway. To this aim, we administered MEK inhibitor U0126 from DIV10 to DIV18 (100 nM, daily) to rNegr1 or control treated neurons. U0126 did not induce overt toxicity as assessed by the incidence of pyknotic nuclei (Supplementary Figures 2A,B). As expected, MEK inhibition significantly impaired neuron morphology. Noteworthy, Negr1 treatment was not able to rescue the morphological changes due to MEK inhibitor both in term of total process length or number (Figures 4A,B, Supplementary Figure 4B, and Table 1). Cumulatively, these data suggest that Negr1 influences neuritic tree outgrowth activating a pathway that requires proper ERK1/2 phosphorylation. It has been demonstrated that IgLON members are able to influence tyrosine kinase associate receptor (RTK; McKie et al., 2012). Furthermore, it is well established that RTK regulate neuronal morphology via the ERK1/2 pathway (Hausott et al., 2009). Consequently, we screened a battery of RTK agonists for their ability to rescue the phenotype associated to Negr1 silencing. Cortical neurons were treated from DIV10 to 18 with IGF (5 ng/ml), FGFb (20 ng/ml), and FGF7 (20 ng/ml). We observed that the treatment with RTK agonists rescued the Negr1 silencing phenotype. Furthermore, we found that FGF7, a specific FGFR2 agonist, induced a more robust increase in neurite number in siNegr1 neurons compared to the other RTK agonists. Similarly, when we administered RTK agonists to control infected cells, only FGF7 treatment was associated to a significant increase in neuritic tree complexity (Figure 5, Supplementary Figure 5, and Table 1). However, when we investigated ERK1/2 pathway activation in neuronal cultures by immuno-blotting, we did not observe any significant increase in ERK1/2 phosphorylation upon chronic treatment with the different agonists (Supplementary Figures 2E,F). To further assess the potential functional correlation among Negr1, FGFR2, and neurite outgrowth, we studied neuron morphology upon acute FGFR2 silencing. To this aim, we took advantage of silencing construct targeting specifically FGFR2 mRNA sequence (Zhou et al., 2006). This construct was able to efficiently down-regulate FGFR2 expression in cortical neurons (Supplementary Figures 2G,H). Cortical neurons were infected at DIV4 with siFGFR2 or control virus, treated or not with soluble rNegr1 at DIV10 and imaged at DIV18. We noticed that FGFR2 silencing was associated with a robust reduction of neuritic tree complexity. Furthermore, we did not observe any increase in neurite number or total length upon rNegr1 treatment in siFGFRF2 neurons (Figures 6A,B, Supplementary Figure 6, and Table 1). Dendritic spines constitute the main postsynaptic elements of excitatory synapses (Tada and Sheng, 2006). Previous reports, including our own, indicated that Negr1 modulated dendritic spine number (Hashimoto et al., 2009; Pischedda et al., 2014). Thus, we imaged dendritic protrusions treated or not with soluble rNegr1. The quantification of spine number and morphology indicated that rNegr1 treatment correlates with an increase in total spine number. To investigate the potential involvement of FGFR2 on this effect, we treated or not with rNegr1 neuronal cultures down-regulated for FGFR2 expression. Interestingly we noticed that FGFR2 silencing did not have an impact on spine number in control treated cultures but abolished the positive effect reported upon Negr1 treatment (Figures 6C,D). We then investigated whether Negr1 can activate the FGFR2 dependent intracellular pathway. To achieve this, siControl and siFGFR2 infected neurons were treated with rNegr1 (40 ng/ml, 10 min) and processed for immuno-blotting to assess the activation of ERK1/2 pathway. These data indicated that acute Negr1 treatment induces ERK1/2 phosphorylation in siControl but not in siFGFR2 neurons (Figures 6E,F). Taken together, these findings suggest that soluble Negr1 influences neuron morphology in a FGFR2 dependent manner.

## Discussion

Our data suggest that Negr1 connects ADAM10 and FGFR2 in a pathway that regulates neurite outgrowth in a ERK1/2 dependent manner. In particular, we provided evidence that ADAM10 shedding releases Negr1 into the extra-cellular space. ADAM10 modulates axonal growth via the cleavage of different membrane proteins including L1-NCAM, N-cadherin, and NCAM (Mechtersheimer et al., 2001; Hinkle et al., 2006; Paudel et al., 2013). We propose that Negr1 acts as a novel ADAM10 substrate involved in neuronal morphogenesis. In a previous study, Sanz and colleagues did not observe any difference in term of Negr1 release upon ADAM10 acute silencing (Sanz et al., 2015). In contrast, in our study we abolished ADAM10 activity pharmacologically via chronic treatment with specific inhibitor. This experimental difference may account for the opposite outcome we reported here.

We propose that soluble Negr1 positively influences neuron maturation by stimulating an intracellular signaling cascade, i.e., acting in trans. Given the outcome we report upon interference with FGFR2 pathway, we suggest that Negr1 triggers ERK1/2 phosphorylation and modulates neurite outgrowth via activation of FGFR2. Accordingly, we describe that acute treatment with rNegr1 triggers ERK1/2 phosphorylation via FGFR2 and that rNegr1 does not influence the morphology of FGFR2 silenced neurons. It is noteworthy, however, that we were unable to detect sustained ERK1/2 phosphorylation upon chronic exposure to rNegr1. Similarly, we did not observe an increase in ERK1/2 phosphorylation upon chronic stimulation with RTK agonists such as IGF, FGFb, or FGF7. The intrinsically transient nature of ERK1/2 signaling might explain this outcome. Alternatively, chronic Negr1 treatment may influence neuronal maturation via a FGFR2 dependent pathway other than ERK1/2 phosphorylation (Mansukhani et al., 2005; Katoh, 2009; Stevens et al., 2010), being in any case ERK1/2 signaling necessary to support proper neurite outgrowth. In any case, chronic treatment with RTK agonist positively influenced the morphology of Negr1 silenced neurons. Thus, RTK agonist and Negr1 may activate a common pathway impinging on ERK1/2 phosphorylation. Interestingly, it is known that FGFR2 signaling via ERK1/2 triggers the phosphorylation and activation of the transcription factor CREB, which modulates neurite outgrowth (Maness and Schachner, 2007). It is tempting to speculate that Negr1 activates a similar signaling cascade. Released Negr1 may stimulate neurite outgrowth via a mechanism similar to the one reported for another ADAM10 substrate, NCAM (Bonfanti, 2006). In fact released NCAM ectodomain stimulates neurite outgrowth triggering ERK1/2 signaling via binding with β1 integrin (Diestel et al., 2005). The data reported here together with our previous publication (Pischedda et al., 2014), show that acute Negr1 down-regulation severely impairs neurite outgrowth. Thus, a neurite outgrowth promoting role for Negr1 *in cis* has to be taken into account. Indeed, we also observed a clear morphological defect upon infection with low titer of Negr1 siRNA viruses. Under these conditions, scarce and isolated Negr1 silenced neurons were surrounded by cells expressing and therefore releasing Negr1. In this experimental setting we observed a clear impairment of neuritic tree complexity upon Negr1 silencing. The magnitude of the effect was, however, lower than that reported upon high viral titer infection, i.e., upon massive and general down regulation of Negr1 protein levels in the cultured neurons. These results suggest that Negr1 influences neuronal development acting both *in cis* and *in trans* and that the two mechanisms contribute to determining neurite outgrowth. We propose that Negr1 may act in trans via FGFR2; however, the precise molecular mechanism underlying Negr1 role *in cis* has yet to be determined. We propose a model where membrane bound Negr1 forms a heterocomplex with FGFR2 and influences its downstream signal upon agonist binding (including potentially soluble Negr1 itself as well as FGFs) as reported for NCAM (Kiselyov et al., 2005; Francavilla et al., 2007, 2009). Indeed Negr1 may not bind directly FGFR2 or instead FGFR2-Negr1 hetero complex is quite unstable, given that we were unable to confirm Negr1-FGFR2 interaction by the means of classic biochemical approaches (data not shown). Several experimental findings connect ADAM10 and FGFR2 to the regulation of neuronal functional and morphological maturation. Indeed, FGFRs play essential roles in almost every step during embryonic brain development (Goetz and Mohammadi, 2013). Axonal path-finding in the visual system and in the peripheral nervous system rely on guidance mechanisms involving FGFRs (Haupt and Huber, 2008). In particular, FGFR2 signaling regulates axon patterning modulating the expression of the repulsive guidance cue Sema3A during innervation (Kettunen et al., 2007). Furthermore, the deletion of FGFR2 results in decreased volume of subcortical white matter due to dramatically reduction number of axonal fibers (Vaccarino et al., 2009). Finally, given that FGFRs signaling has a prominent role in the maturation and migration of neurons (Smith et al., 2006; Stevens et al., 2010; Müller Smith et al., 2012), it is tempting to speculate about a putative role for Negr1 in overall CNS development. Taken together, our data suggest a possible functional link among Negr1, ADAM10, and FGFR2. It is also of note that all three of these proteins have been linked to different neurological disorders. Independent genetic studies have linked both Negr1 and FGFR2 to autism (Pinto et al., 2010; Hussman et al., 2011; Casey et al., 2012; Michaelson et al., 2012; Neale et al., 2012). Although the mechanisms that lead to autism are at best poorly understood, recent findings on neurological functioning point to altered brain connectivity as a key feature in this disorder (Vissers et al., 2012; Coskun et al., 2013; Schaer et al., 2013). Alteration of Negr1 expression has been described in two other neurological disorders characterized by marked connectivity dysfunctions, namely dyslexia (Veerappa et al., 2013) and schizophrenia (Holliday et al., 2009), suggesting that Negr1 plays pivotal role in the establishment of a functional wiring. Finally, altered levels of Negr1 have been observed in the CSF of psychiatric patients, raising the possibility that soluble Negr1 may be pathologically relevant (Maccarrone et al., 2013). Finally, ADAM10 has been implicated in the pathogenesis of Alzheimer's disease (AD): ADAM10 possesses alpha-secretase activity, involved in the alternate amyloid precursor protein processing pathway to the beta-secretase pathway that produces amyloid beta (Lammich et al., 1999). Two rare ADAM10 variants, Q170H, and R181G, have been correlated with AD and result in defective alpha-secretase activity (Kim et al., 2009; Suh et al., 2013). It would be interesting to study whether Q170H and R181G mutations impair also Negr1 shedding and eventually evaluate the potential role of soluble Negr1 in such models. In conclusion, our data show that Negr1 has the ability to influence neuronal signaling during neuronal network formation acting both as a membrane bound protein as well as a soluble factor. Given its possible implication with several neurological disorders, clarifying the function of Negr1 and the underlying biological pathways linked to neuronal function may provide a potential target for future therapeutic intervention.

## Author contributions

FP performed and designed experiments; GP designed experiments and wrote the paper.

### Conflict of interest statement

The authors declare that the research was conducted in the absence of any commercial or financial relationships that could be construed as a potential conflict of interest.

## Supplementary material

Supplementary Figure 1 **Cortical neurons were infected with siControl virus at DIV4 and treated every second day from DIV10 to DIV18 with DMSO (not treated, NT) or with ADAM10 inhibitor GI 254023X (20 μM) and/or recombinant Negr1 (40 ng/ml, single administration at DIV 10, rNegr1)**. Neurons were processed for immunofluorescence at DIV18 and GFP positive neurons imaged via confocal microscopy. Graph shows the number of neurite belonging to 1st, 2nd, 3rd, and 4th order. Data are reported as mean ± SEM; ^*^*p* < 0.01 vs. not treated, °*p* 0.001 vs. GI 254023X.

Supplementary Figure 2 **Cortical neurons were treated daily from DIV10 to DIV18 every second day with GI 254023X (GI, 20 μM) or MEK inhibitor U0126 (U0126, 100 nM)**. At DIV18 cells were processed for imaging and stained with DAPI to detect nuclei. The presence of pyknotic/condensed nuclei (indicated with an arrow) reveals cell toxicity. Scale bar = 50 μm **(A)**. The graph reports the percentage of pyknotic nuclei. Data are expressed as mean ± SEM **(B)**. Recombinant Negr1 (rNegr1) was purified on streptavidin resin from transfected HEK293 cells. Protein purity was assessed by SDS-PAGE followed by silver staining. (**C**, left). The plot indicates optical density measured along the gel lane, expressed in arbitrary unit (**C**, right). rNegr1 was treated or not with PNGase (5 units, 20 min, 37°C) and then analyzed by western blotting. Fully glycosylated rNegr1 runs at the apparent molecular weight of 50 kDa **(D)**. Cortical neurons were treated daily from DIV10 to DIV18 with IGF (5 ng/ml), FGFb (20 ng/ml), and FGF7 (20 ng/ml) or with rNegr1 (40 ng/ml, single administration at DIV10). At DIV18 cells were processed for western-blotting analysis. We did not observe a clear induction of ERK1/2 phosphorylation upon treatment with the different molecules **(E)**. The graph reports p-ERK1/2 level normalized vs. total ERK1/2 amount. Data are expressed as mean ± SEM *N* = 5 **(F)**. Cortical neurons were infected at DIV4 with virus expressing siControl or siRNA against FGFR2 (siFGR2) and processed for western-blotting at DIV18 **(G)**. The graph reports the optical density of the band relative to FGFR2, normalized vs. actin value. Data are reported as mean ± SEM; *N* = 5, ^*^*p* < 0.001 vs. siControl **(H)**.

Supplementary Figure 3 **The figures report the original images acquired at the microscope (upper lane), the images superimposed with the relative neurite tracing elaborated on NeuronJ (middle lane) and the tracings alone as they appear in the main figures (lower lane)**. Panels are 202 × 202 μm large. Supplementary Figure 3B (not treated panel) exemplifies the output of the software assisted neurite analysis: counted neurites are indicated by a progressive number. The correlation among main and Supplementary Figures is: Figure 1 **=** **Supplementary Figure 3A**; Figure 2 **=** **Supplementary Figure 3B**; Figure 3 **=** **Supplementary Figure 4A**; Figure 4 **=** **Supplementary Figure 4B**; Figure 5 **=** **Supplementary Figure 5**; Figure 6 **=** **Supplementary Figure 6**.

Supplementary Table 1 **The table reports the complete outcome of the statistical analysis; ^*^***p*** < 0.05, ^**^***p*** < 0.01, and ^***^***p*** < 0.001**.
