# Supplementary figures and images for: The IgLON Family Member Negr1 Promotes Neuronal Arborization Acting as Soluble Factor via FGFR2
Source: Front Mol Neurosci. 2016 Jan 13;8:89. doi: 10.3389/fnmol.2015.00089 (PMC4710852; doi:10.3389/fnmol.2015.00089)

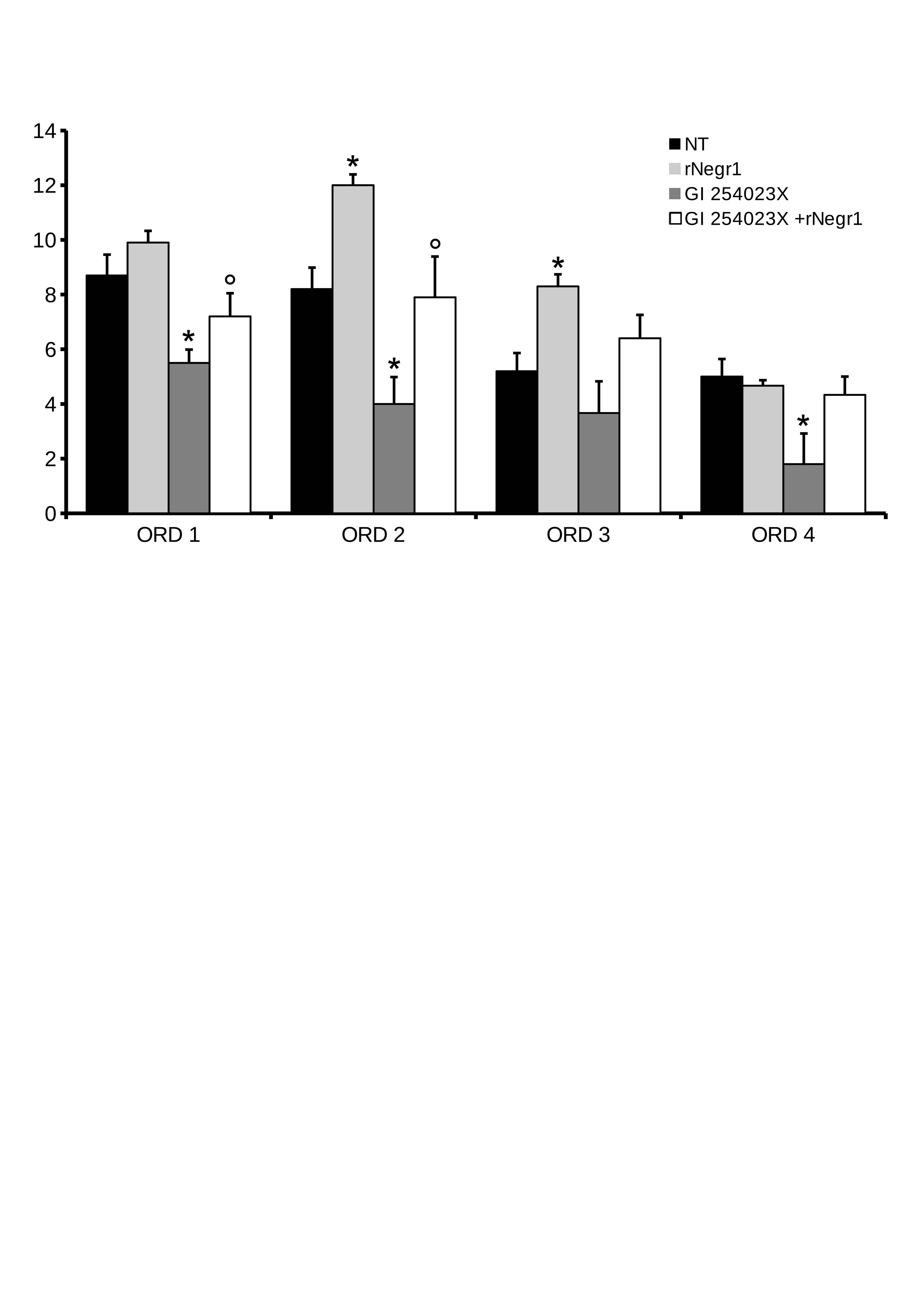

Supplement: Supplementary Figure 1 — Cortical neurons were infected with siControl virus at DIV4 and treated every second day from DIV10 to DIV18 with DMSO (not treated, NT) or with ADAM10 inhibitor GI 254023X (20 μM) and/or recombinant Negr1 (40 ng/ml, single administration at DIV 10, rNegr1). Neurons were processed for immunofluorescence at DIV18 and GFP positive neurons imaged via confocal microscopy. Graph shows the number of neurite belonging to 1st, 2nd, 3rd, and 4th order. Data are reported as mean ± SEM; *p < 0.01 vs. not treated, °p 0.001 vs. GI 254023X. [file Image1.JPEG]

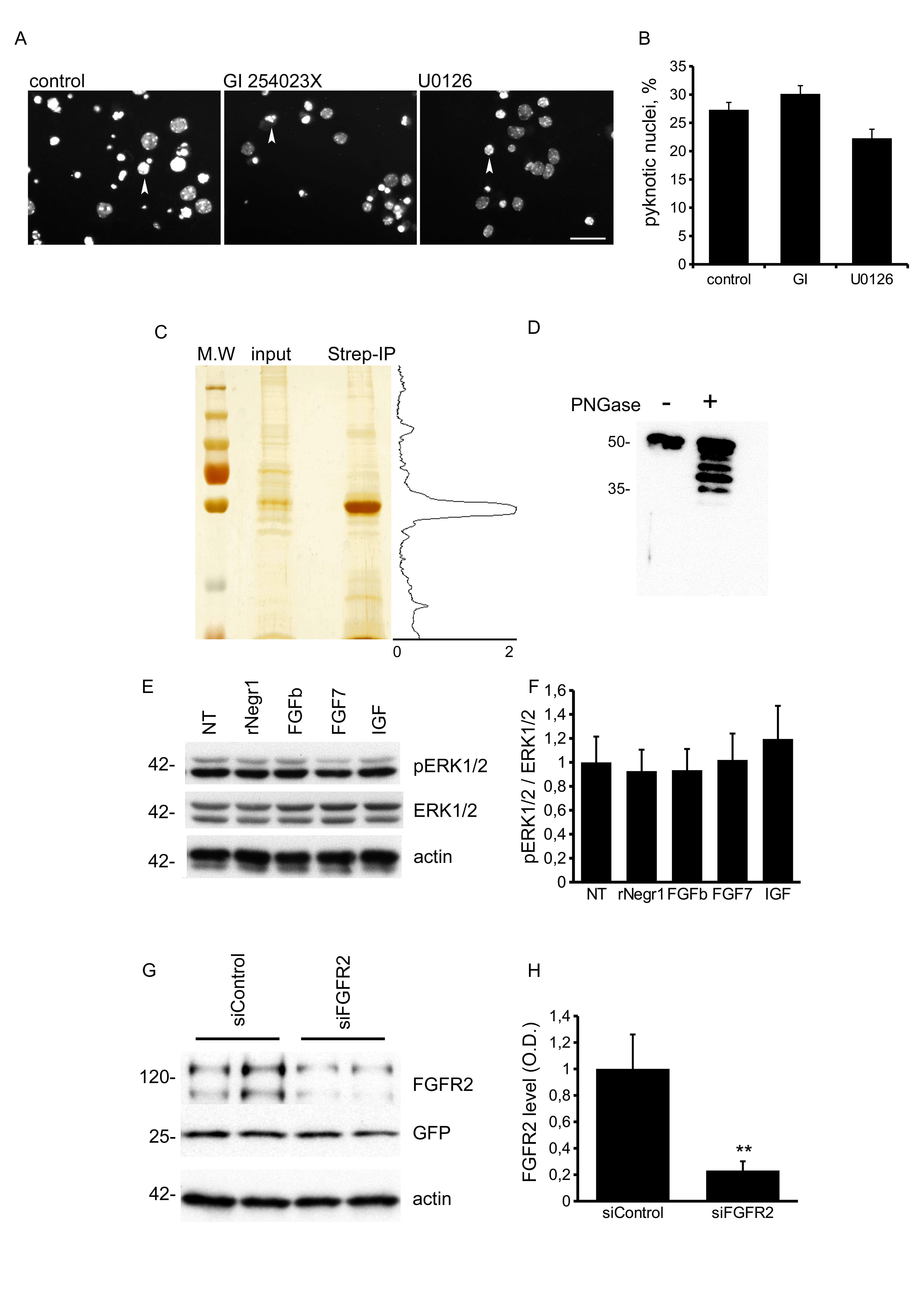

Supplement: Supplementary Figure 2 — Cortical neurons were treated daily from DIV10 to DIV18 every second day with GI 254023X (GI, 20 μM) or MEK inhibitor U0126 (U0126, 100 nM). At DIV18 cells were processed for imaging and stained with DAPI to detect nuclei. The presence of pyknotic/condensed nuclei (indicated with an arrow) reveals cell toxicity. Scale bar = 50 μm (A). The graph reports the percentage of pyknotic nuclei. Data are expressed as mean ± SEM (B). Recombinant Negr1 (rNegr1) was purified on streptavidin resin from transfected HEK293 cells. Protein purity was assessed by SDS-PAGE followed by silver staining. (C, left). The plot indicates optical density measured along the gel lane, expressed in arbitrary unit (C, right). rNegr1 was treated or not with PNGase (5 units, 20 min, 37°C) and then analyzed by western blotting. Fully glycosylated rNegr1 runs at the apparent molecular weight of 50 kDa (D). Cortical neurons were treated daily from DIV10 to DIV18 with IGF (5 ng/ml), FGFb (20 ng/ml), and FGF7 (20 ng/ml) or with rNegr1 (40 ng/ml, single administration at DIV10). At DIV18 cells were processed for western-blotting analysis. We did not observe a clear induction of ERK1/2 phosphorylation upon treatment with the different molecules (E). The graph reports p-ERK1/2 level normalized vs. total ERK1/2 amount. Data are expressed as mean ± SEM N = 5 (F). Cortical neurons were infected at DIV4 with virus expressing siControl or siRNA against FGFR2 (siFGR2) and processed for western-blotting at DIV18 (G). The graph reports the optical density of the band relative to FGFR2, normalized vs. actin value. Data are reported as mean ± SEM; N = 5, *p < 0.001 vs. siControl (H). [file Image2.JPEG]

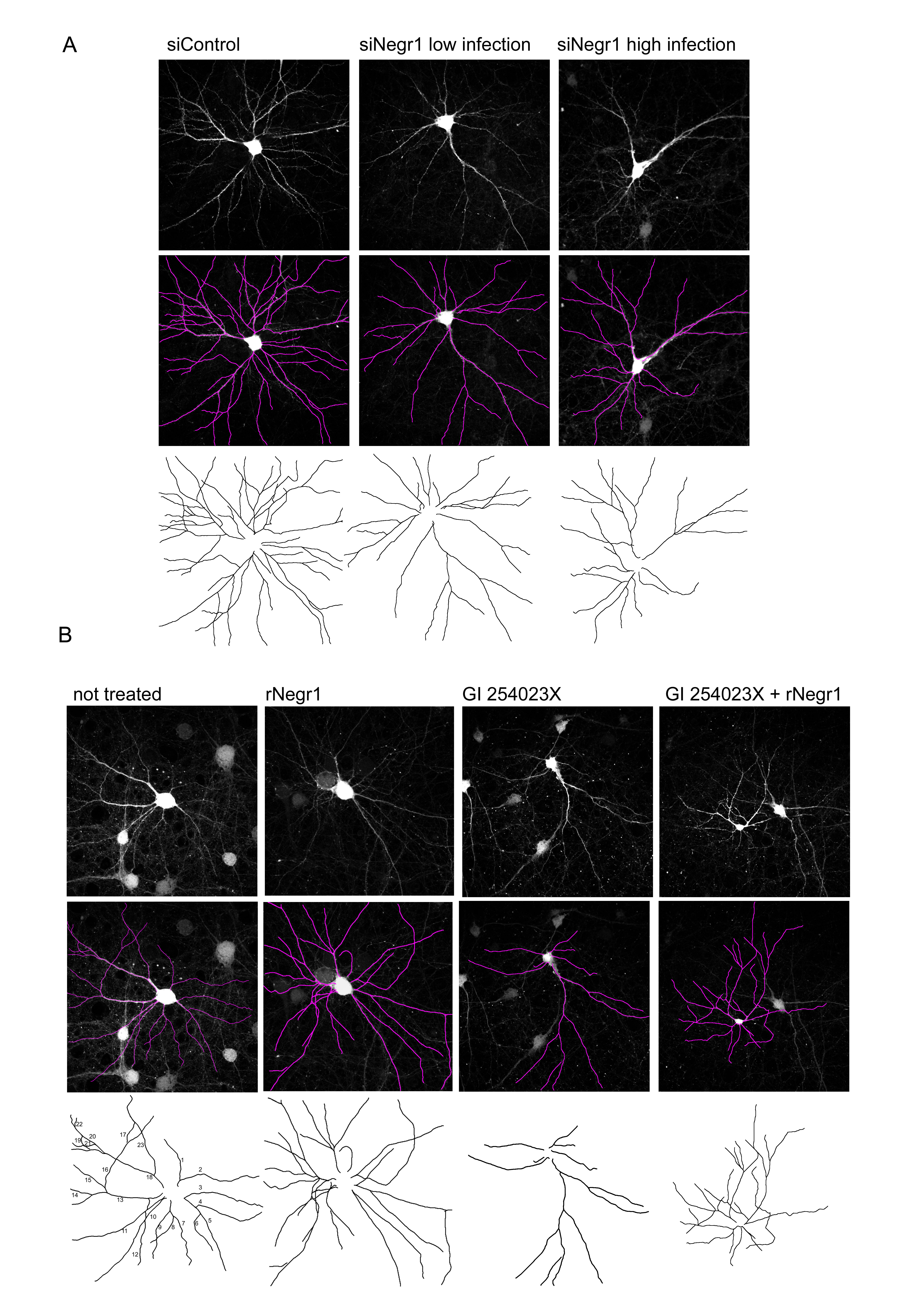

Supplement: Supplementary Figure 3 — The figures report the original images acquired at the microscope (upper lane), the images superimposed with the relative neurite tracing elaborated on NeuronJ (middle lane) and the tracings alone as they appear in the main figures (lower lane). Panels are 202 × 202 μm large. Supplementary Figure 3B (not treated panel) exemplifies the output of the software assisted neurite analysis: counted neurites are indicated by a progressive number. The correlation among main and Supplementary Figures is: Figure 1 = Supplementary Figure 3A; Figure 2 = Supplementary Figure 3B; Figure 3 = Supplementary Figure 4A; Figure 4 = Supplementary Figure 4B; Figure 5 = Supplementary Figure 5; Figure 6 = Supplementary Figure 6. [file Image3.JPEG]

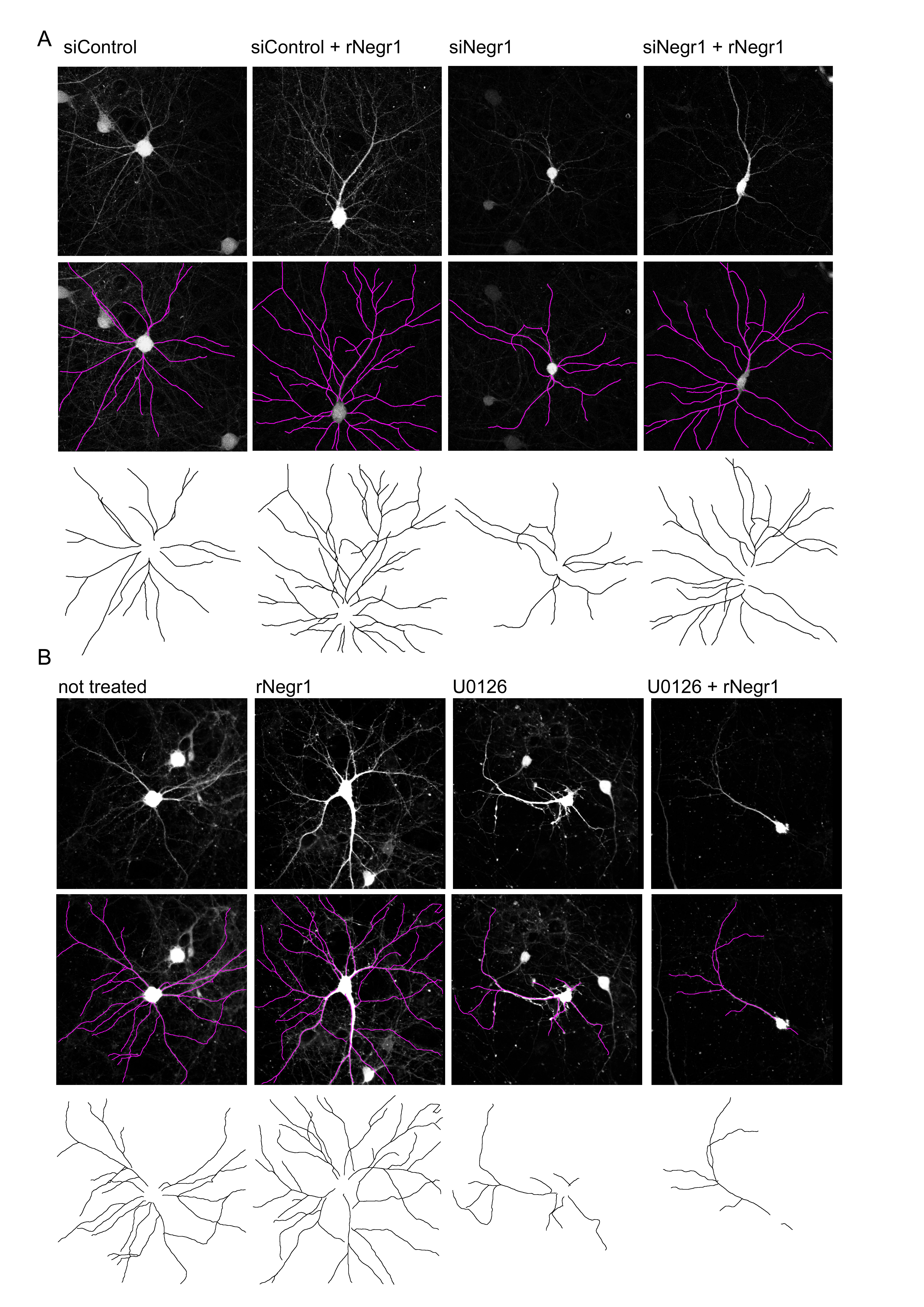

Supplement: Supplementary file 4 [file Image4.JPEG]

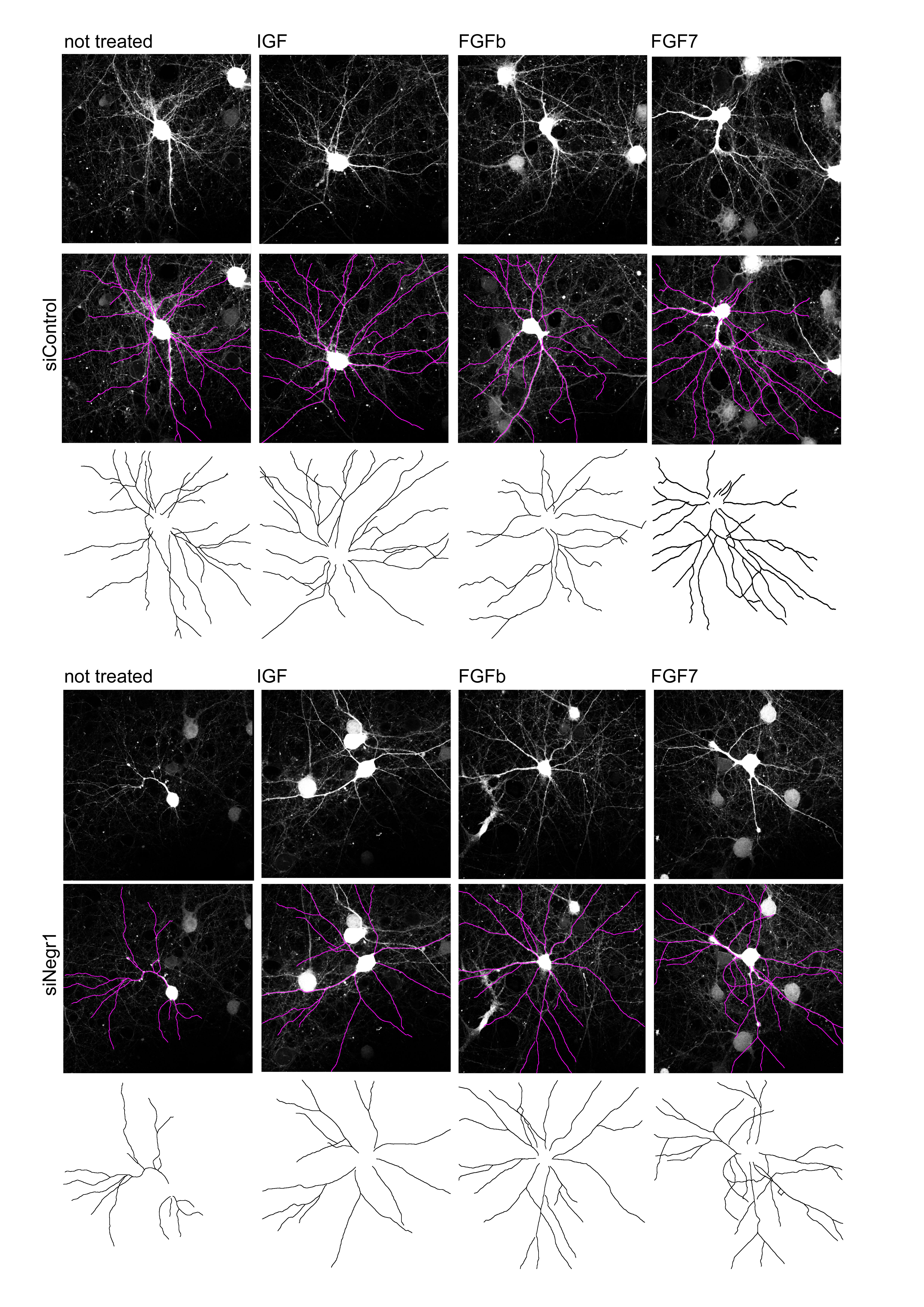

Supplement: Supplementary file 5 [file Image5.JPEG]

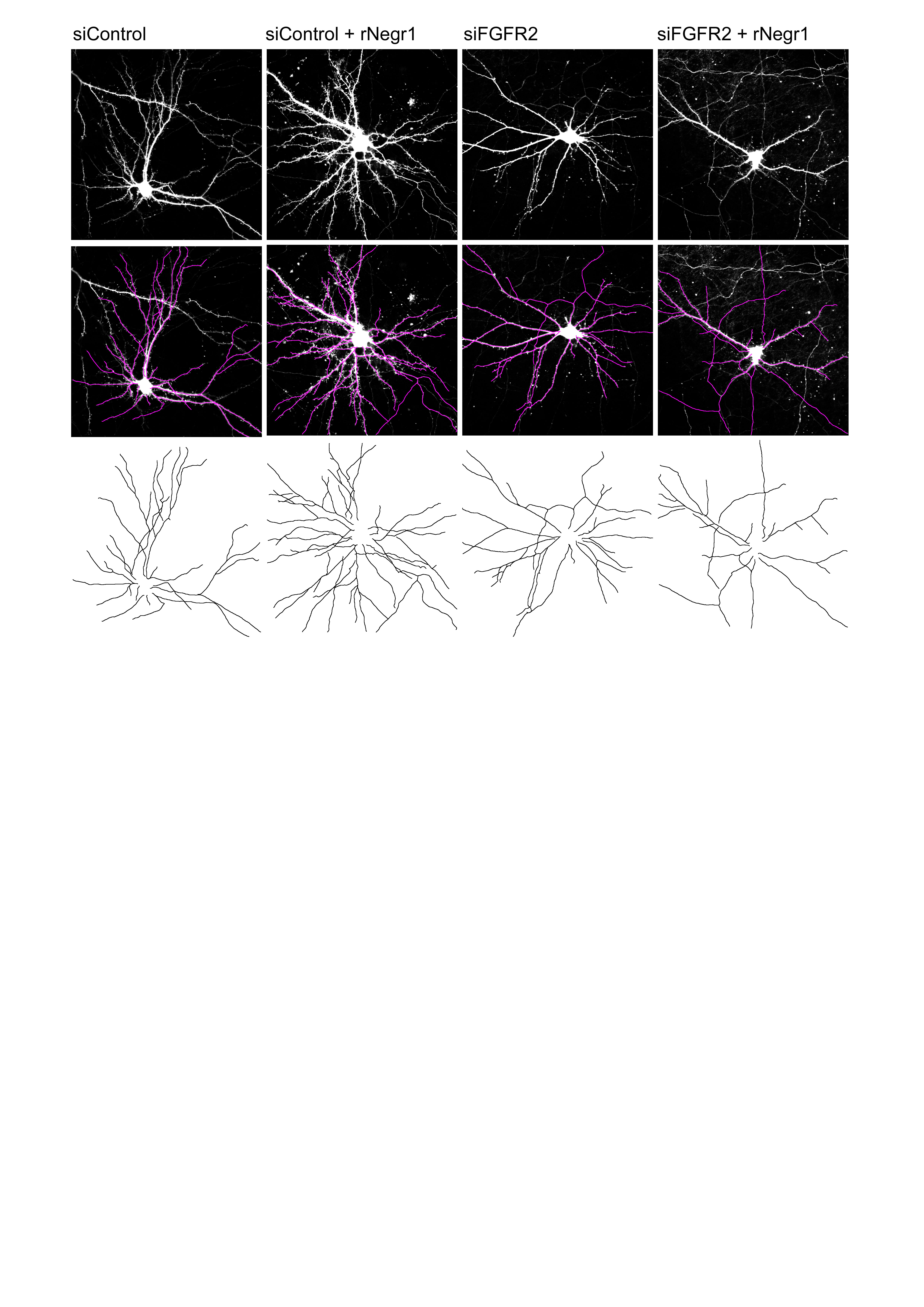

Supplement: Supplementary file 6 [file Image6.JPEG]
